# *Mbd3*, a Component of NuRD/Mi-2 Complex, Helps Maintain Pluripotency of Mouse Embryonic Stem Cells by Repressing Trophectoderm Differentiation

**DOI:** 10.1371/journal.pone.0007684

**Published:** 2009-11-03

**Authors:** Dongmei Zhu, Junshun Fang, Yanxin Li, Jian Zhang

**Affiliations:** 1 Laboratory of Molecular and Developmental Biology, Institute of Genetics and Developmental Biology, Chinese Academy of Sciences, Beijing, China; 2 Graduate School of the Chinese Academy of Sciences, Beijing, China; Baylor College of Medicine, United States of America

## Abstract

Embryonic stem cells (ES cells) can differentiate into cells derived from all three germ layers and extraembryonic tissues. While transcription factors such as, *Oct4* and *Nanog* are well known for their requirements for undifferentiated ES cell growth, mechanisms of epigenetic repression of germ layer specific differentiation in ES cells are not well understood. Here, we investigate functions of Mbd3, a component of nucleosome remodeling and histone deacetylation complex (NuRD/Mi-2) in mouse ES cells. We find that compared to wild type ES cells, *Mbd3* knockdown cells show elevated RNA expression of trophectoderm markers, including *Cdx2, Eomesodermin, and Hand1*. In parallel, these cells show an increased acetylation level of histone 3 in promoters of the respective genes, suggesting *Mbd3* plays a role in repression of these genes in undifferentiated ES cells. However, these changes are not sufficient for definitive differentiation to trophectoderm (TE) in chimeric embryos. When further cultured in ES medium without LIF or in trophoblast stem (TS) cell medium, *Mbd3* knockdown cells differentiate into TE cells, which express Cdx2 and, at later stages, trophoblast lineage specific marker Cadherin 3. These results suggest that *Mbd3* helps restrict ES cells from differentiating towards the trophectoderm lineage and is an important epigenetic player in maintaining full pluripotency of mouse ES cells.

## Introduction

Embryonic stem (ES) cells are derived from the inner cell mass (ICM) of growing blastocysts. They maintain an undifferentiated state in defined culture conditions, but can also be induced to differentiate into diverse cell types representative of all three germ layers both in vitro and in vivo [1]. ES cells are powerful tools for expanding our knowledge in mammalian early development and are thought to hold great promise for regenerative medicine [2]. ES cells share many characteristics of ICM cells at the level of transcriptional regulation. For example, they both express pluripotent cell specific transcription factors, such as *Oct4* and *Nanog* [3]–[5]. In mouse, loss of *Oct4* expression by targeted gene deletion causes ES cells to develop into trophectoderm [6], [7], while deletion of *Nanog* causes ES cells to differentiate into primitive endoderm [5] and to compromise PGC maturation [8]. Considerable efforts have been devoted to elucidate transcriptional networks of these and other transcription factors and their associated cofactors [9], [10]. These transcription factors have been implicated in cooperatively activating or repressing a broad range of downstream target genes [11]. However, less attention has been paid to epigenetic regulation of these lineage specific transcription factors. Recent studies have shown that the ES cell pluripotent state is critically maintained by Polycomb group (PcG) complexes that mediate suppression of key differentiation genes [12]–[14]. Other epigenetic studies point to similar lineage restriction schemes to govern ES cell pluripotency (reviewed in [15]). Despite these studies, detailed mechanisms of how global epigenetic control is achieved, especially how lineage specific transcription programs are suppressed in ES cells, remain to be fully elucidated (reviewed in [15], [16]).

Major epigenetic modifications include DNA methylation, histone acetylation and methylation which are often closely coupled [17]. DNA methylation at the dinucleotide CpG in regulatory regions is a hallmark of stable transcriptional silencing [18]. Recruitment of specific binding proteins to methylated CpG islands is believed to repress target gene transcription [19]. On the other hand, acetylation of histone tails is critical for nucleosome structure alterations that facilitate DNA accessibility to regulatory factors [20]–[22].

Purification of nucleosome remodeling and histone deacetylation complex (NuRD, also known as Mi-2, NURD, or NRD) links together two epigenetic modifications: DNA methylation and histone deacetylation [23]–[27]. Several components of the NuRD complex have been shown to be necessary for early embryonic development. Methylated DNA-linked chromosomal remodeling and gene silencing are thought to be mediated by methyl-CpG binding (MBD) proteins [19], [28]. Unlike other mammalian MBD protein, Mbd3 does not bind to methyl-CpG biochemically. Instead, Mbd3 is directly associated with Chd4 protein as core subunits of the NuRD complex. Study of *Mbd3* null mice indicates that it is essential for early embryogenesis while *Mbd2* is dispensable for viability [29]. Since dynamic epigenetic regulations occur during ICM formation and differentiation of primary germ layers, early embryonic lethality caused by *Mbd3* deletion may be attributed to abnormal epigenetic modifications, and therefore dysregulation of gene expression in early embryos [30], [31]. *Mbd3* function was reported to be dispensable for ES cell growth in culture, but essential for their commitment to a full spectrum of embryonic lineages when aggregated with wild type embryos, indicating pluripotency of these cells is indeed affected [15], [32]. A detailed mechanism for restricted differentiation of the *Mbd3*-deficient cells remains to be elucidated. Interestingly, when cultured in vitro to promote embryonic stem cell outgrowth, *Mbd3*-deficient ICMs fail to generate pluripotent cells [33]. This difference may be attributed to different sets of molecular factors that are required for the derivation and maintenance of the pluripotent state [15], [33].

Specification of trophectoderm is the first sign of differentiation of early mouse embryos. Studies of molecules required for the specification of the trophectoderm have led to identification of Oct4 as a negative regulator while Cdx2 as a positive transcription factor in the process. Conditional deletion of *Oct4* in mouse ES cells leads to trophoblast differentiation and increased expression of trophoblast-specific markers [6]. Trophoblast stem (TS) cells can be derived when these cells are cultured under conditions that promote trophoblast proliferation [34]. On the other hand, Cdx2 is specifically expressed in outer cells of the blastocyst, which are destined to form trophectoderm [35]. Without functions of Cdx2, transcription of *Oct4* and *Nanog* are not downregulated in these outer cells, thus resulting in the implantation failure of the mutant embryos [35].

Interestingly, though *Cdx2* is essential for TS cell self-renewal, it is dispensable for trophectoderm differentiation induced by Oct4 repression [34]. Since Cdx2 and Oct4 form a complex in early embryos, reciprocal inhibition of their respective target genes was proposed to be important in achieving the correct segregation of the ICM and trophectoderm lineages [34], [36].

Although studies using *Mbd3* ^−/−^ ES cells have greatly helped us to understand the roles of NuRD complex in maintaining full ES pluripotency, the underlying molecular mechanism remains obscure. In the present study, we selectively reduced expression of *Mbd3* in mouse ES cells by RNA interference to address why *Mbd3* is required for maintain mouse ES cell pluripotency. We find that reduction of *Mbd3* compromises the full differentiation potential of ES cells. Moreover, with reduced *Mbd3* expression, mouse ES cells are set at an intermediate state and are more prone to differentiate into trophectoderm. Our results suggest that *Mbd3* is involved in maintaining pluripotency of mouse ES cells by repressing trophectoderm differentiation.

## Materials and Methods

### Plasmids

Mbd3 and its control short hairpin RNA (shRNA) plasmids were all placed into pSuper.retro.puro vector (Oligo Engine Inc.). RNA interference (RNAi) target sequences for *Mbd3* were selected using Ambion siRNA converter online software (http://www.ambion.com/techlib/misc/siRNA_finder.html). The target sequences are as follows:

*Mbd3* shRNA1: 5′-GATGAATAAGAGTCGCCAG-3′

*Mbd3* shRNA2: 5′-AGCCTTCATGGTGACAGAT-3′;

*Mbd3* control shRNA: 5′-GCGAAGTGCATTGTGTGGC-3′.

Oligonucleotides were annealed and inserted into Bgl II/HindIII sites of pSuper.retro.puro vector. EGFP fragment from pEGFP-N1 (Clontech) was subcloned into the AccI site of the RNAi plasmids to visualize transfected cells.

Mouse *Mbd3* cDNA and full-length human *Mbd3* cDNA were cloned into XbaI and HindIII sites in pRK5-tkneo vector (Genentech, South San Francisco, Calif. [37]).

### Cell culture, plasmid transfection and cell proliferation assay

Mouse ES cell line CGR8 (kindly provided by Dr. Austin Smith) [6] was maintained in GMEM (Sigma G5154) supplemented with 10% fetal bovine serum (PAA, pre-tested for ES cells, A15-080), 1 mM sodium pyruvate (Sigma S8636), 2 mM L-glutamine (Hyclone SH30034), 0.1 mM non-essential amino acids (Hyclone SH30238), 0.1 mM 2-mercaptoethanol (Sigma M7522), 50 µg/ml penicillin/streptomycin (Hyclone SV30010), 10^3^ Units/ml leukemia inhibitory factor (LIF, Chemicon ESG1107). CGR8 cells were cultured in plates coated with 0.1% gelatin (Sigma G9391) without feeder layer cells. NIH3T3 and mouse primary fibroblast cells were maintained in DMEM (Hyclone SH30022) supplemented with 10% fetal bovine serum (Hyclone SH30088), 50 µg/ml penicillin/streptomycin, and 2 mM L-glutamine.

Plasmids were transfected into cells with PolyFect (Qiagen 301105) or Lipofectamin 2000 (Invitrogen 11668) according to the manufactures' instructions. All antibiotic selections were started at 24 hours after transfections. Mouse CGR8 ES cells were selected with 1 µg/ml puromycin (Sigma P8833) and/or 300 µg/ml G418 (Invitrogen 11811-023).

For cell proliferation assays, transfected cells were maintained in medium without antibiotic selection. Equal numbers of EGFP positive cells were seeded in triplicate in 12-well plates one day after transfection. Green fluorescent cells were counted in the following days. Each experiment was repeated at least three times.

### Lentivirus construction, package and infection

For generation of ES cell lines stably overexpressing shRNA, the oligonucleotides used for *Mbd3* shRNA1 were cloned into pLentilox3.7 vector. For lentivirus production, pLentilox3.7 plasmids were co-transfected with packaging vectors into 293T cells, and the supernatant was harvested after 48 hours. After centrifugation and filtration, the supernatant was added into ES cell suspension for infection. Single colonies were then picked and propagated.

### Western blotting

Cells were collected after trypsinization and washed twice with cold PBS. Cell lysate was extracted with five times volume of cold EBC buffer (120 mM NaCl, 50 mM Tris-Cl PH 8.0, 0.5% Nonidet P-40) containing protease inhibition cocktails (Roche 1697498) and 1 mM PMSF (Amersco 0754). Protocols used for protein fractionation in SDS-PAGE, blotting and antibody incubation were essentially the same as those described in Molecular Cloning [38]. Anti-MBD3 (C-18) (sc-9402) was purchased from Santa Cruz and anti-α-Tubulin (T5168) antibodies was from Sigma. The protein signals were detected using Pierce SuperSignal kit (Pierce 34095) and chemiluminescent images were captured using a cold CCD camera (UVP BioImaging Systems).

### Real-time RT-PCR

Total RNAs were extracted with Trizol reagent (Invitrogen 15596-026) followed by DNase I (Roche 11994020) treatment. Reverse transcription reactions from 2 µg RNA were carried out with MMLV reverse transcriptase (Invitrogen 28025-013). cDNAs from 25 ng of RNA were used as templates for quantitative PCR amplification using SYBR PCR Master Mix (ABI 4367659) in ABI Prism 7900 HT sequence detection system (Applied Biosystems). Data were analyzed by SDS2.2 software. Reactions were set up in triplicate for each sample. Gene expressions were normalized to *β-actin* expression. Data are shown as fold inductions relative to control. Primers are shown in Table S1.

### Chromatin immunoprecipitation (ChIP)

About 10^6^ cells for ChIP were treated with shRNA for six days while selected with puromycin for five days. ChIP assays with an acetylated histone 3 (AcH3) antibody (Upstate, Catalog # 17–245) were carried out following the manufacture's protocol. Briefly, cells were cross-linked with 1% formaldehyde for 10 min at 37°C. Cell lysates were sonicated at 100 w for 10 s with ultrasonic apparatus (Scientz JY92-2D). The sonication step was repeated four times with 30 s intervals. Chromatin extracts containing DNA fragments with an average size of 500 bp were immunoprecipitated using 5 µg AcH3 antibody. Quantitative PCR was carried out as described in real-time RT-PCR section. Primers are shown in Table S2.

### Embryoid body (EB) formation and chimeric embryo production

For EB formation assays, 2×10^5^ cells were seeded into 35 mm low attachment sterile cell plate (Ai Si Jin Co., China) in 2 ml ES cell medium without LIF. Fresh medium was exchanged every two days.

For chimera production, eight-cell stage embryos were collected from ICR female mice. Embryos were treated with acidified Tyrode's solution (Sigma T1788) for 10 sec. to remove the zona pellucida. Naked embryos were washed through four droplets of M2 medium (Sigma M7167) and subsequently cultured in the “well-in-well” of 50 µl KSOM-AA medium (Chemicon MR-106-D) individually to maintain the developmental competency and embryonic integration. One small cluster of *Mbd3* knockdown stable cells (10–20 cells) was gently put into the culture droplet which contained the naked embryo. After 24 h, either aggregated morula or blastocyst stage embryos were selected. Morula-stage embryos were further cultured in 50μl droplet of fresh KSOM medium until the blastocyst stage [39].

### Trophoblast stem (TS) cell derivation and cell immunostaining

CGR8 cells were treated with Mbd3 shRNAs for at least three days then cultured in TS cell culture condition. TS cells were derived and maintained in 30% fresh TS medium [GMEM (Sigma G5154) supplemented with 20% (v/v) of FBS (Hyclone SH30396), 1 mM sodium pyruvate (Sigma S8636), 2 mM L-glutamine (Hyclone SH30034), 0.1 mM 2-mercaptoethanol (Sigma M7522), 50μg/ml penicillin/streptomycin (Hyclone SV30010), 1μg/ml of sodium heparin (Sigma H3149), and 25 ng/ml of recombinant FGF4 (Sigma F8424)] and 70% (v/v) of the MEF-conditioned TS medium [40]. MEF-conditioned medium was collected from mitomycin C-treated MEF cells cultured in TS medium for 3 days.

For immunostaining, cells were fixed in 4% paraformaldehyde at room temperature for 10 min. After rinsing twice with PBS, cells were blocked with blocking buffer (PBS+0.1% Gelatin+1% BSA+0.02% NaN3+0.4% TritonX-100) for 30 min at room temperature (for Cdh3 staining, withdrawal of TritonX-100 from blocking buffer). Primary antibodies anti-Cdh3 (Neomarkers, MS-1741) and anti-Cdx2 (BioGenex, MU392-UC) were diluted at 1∶100 and 1∶50 in blocking buffer, respectively. Secondary antibody detecting mouse IgG conjugated with TRITC or FITC (ZhongShanJinQiao, ZF-0313, 0312) were diluted at 1∶100 in blocking buffer. Fixed cells were stained with primary and secondary antibodies for 1 hour, respectively. Hoechst (Sigma B2261) were used for cell nuclei staining. Images were captured with a fluorescence microscope (Nikon Eclipse TE2000-U) or Zeiss confocal microscope (LSM510META).

## Results

### *Mbd3* is required in mouse ES cells for suppressing expression of trophectoderm specific genes

Much progress has been made in defining requirements for maintenance and differentiation of ES cells, but only limited information is available as to how lineage restriction is achieved epigenetically [41]. One of the major epigenetic regulations is modulation of histone acetylation levels at genes required for certain biological processes. The NuRD complex uniquely processes both nucleosome remodeling and histone deacetylase activities and functions primarily in transcriptional repression [42]. To better understand the functions of the NuRD complex in maintaining pluripotency of mouse ES cells, we investigated its functional components. We chose to inhibit expression of *Chd4* (will be reported elsewhere) and *Mbd3* which is essential for very early development as demonstrated in mouse *Mbd3* knock-out experiments [29]. More recent studies by Kaji and colleagues strongly suggest that *Mbd3* is critically required for mouse embryonic stem cells both in vitro and in vivo [32], [33]. However, the underlying mechanisms for the requirement remain to be fully elucidated.

**Figure 1:**
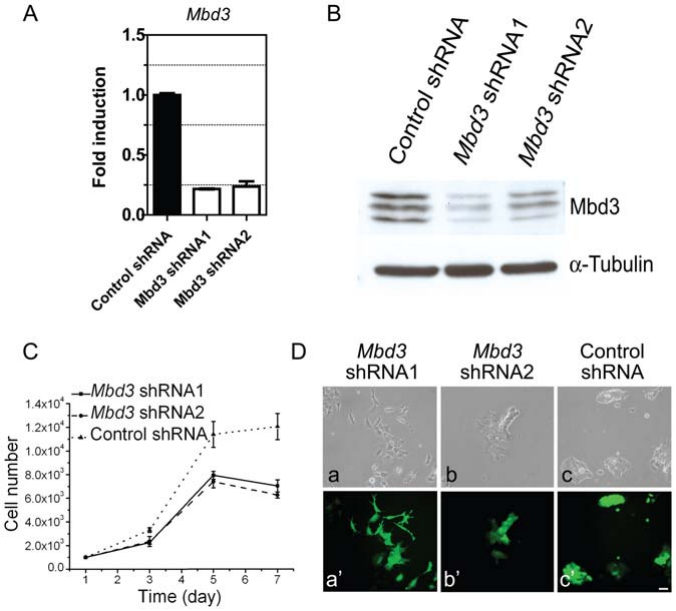
Reduced *Mbd3* expression in CGR8 cells results in morphological differentiation. A) Quantitative RT-PCR analysis *Mbd3* mRNA expression in *Mbd3* shRNA transfected CGR8 cells, which were selected with puromycin for five days. Gene expressions are normalized to internal control *β* -actin and presented as the fold induction relative to control shRNA. Error bars represent standard deviation from three technical repeats. B) *Mbd3* shRNA transfections downregulates endogenous Mbd3 protein expression in CGR8 cells detected on western blots. Cells were transfected with indicated shRNA plasmids. C) Growth curves of CGR8 cells transfected with shRNA plasmids. Only GFP positive cells were counted. Error bars represent standard deviation. D) CGR8 cells were transfected either with control shRNA(a, a′) or *Mbd3* shRNA1/2 (b, b′ and c, c′) and selected for five days with puromycin. GFP fluorescence in a′, b′, c′ indicate that the cells harbor shRNA plasmids. The scale bar represents 50μm.

Two RNA interference plasmids were made against mouse *Mbd3*. Transfection of either shRNA plasmids can efficiently reduce both *Mbd3* RNA expression and its protein expression in CGR8 cells (Figure 1A, B). We first asked if *Mbd3* shRNAs treatment influences ES cell proliferation. *Mbd3* shRNAs transfected cells (thereafter referred as *Mbd3* shRNA cells) showed only a slightly lower proliferation rate compared with wild type ES cells (Figure 1C). However, the majority of the *Mbd3* shRNA cells displayed marked differentiated morphology when compared to control shRNA cells. In contrast to tightly packed ES cell colonies with smooth edges, *Mbd3* shRNA cells showed morphological changes ranging from a fibroblast-like shape to loosely associated cell aggregations (Figure 1D, compare a,a′ to b,b′ and c,c′). Minor cell proliferation changes after *Mbd3* shRNA transfection may be partially explained by ES cell differentiation into other cell types. These results indicate that *Mbd3* may be essential for maintaining mouse ES cells in an undifferentiated state.

**Figure 2:**
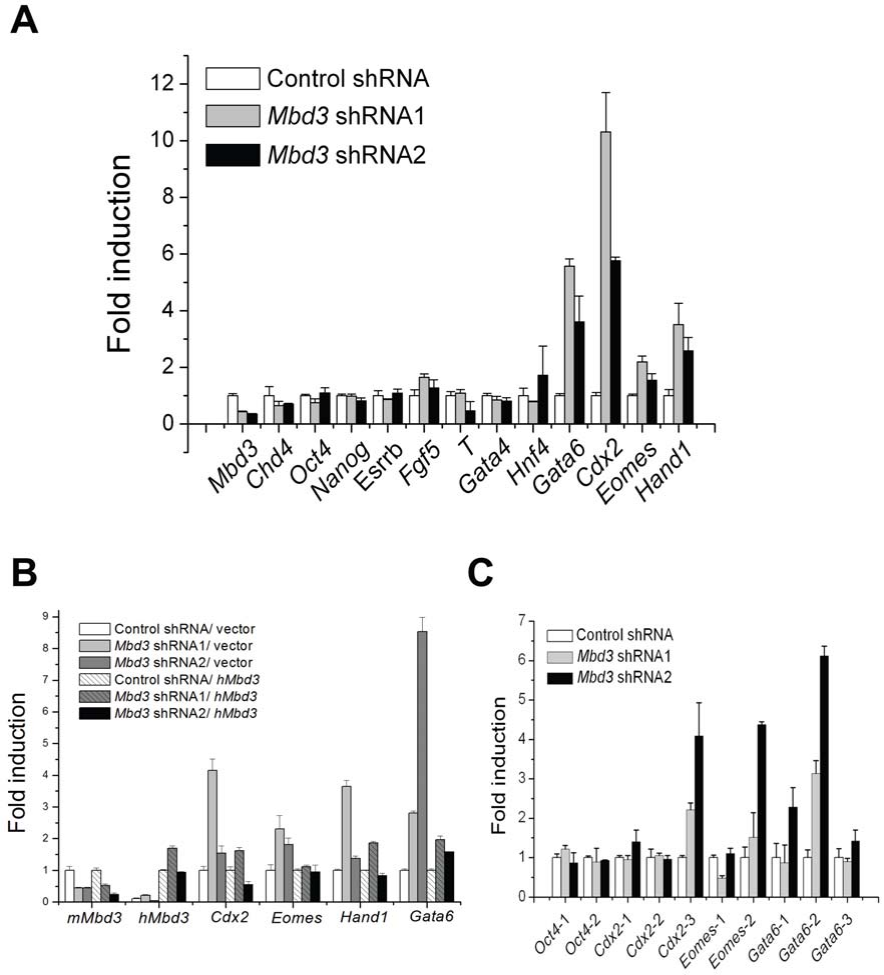
Suppression of Mbd3 results in the upregulated gene expressions of trophectoderm lineage markers in CGR8 cells. Equal amounts of DNase-treated total RNA were subjected to quantitative RT-PCR analysis (A, B). A) Cells were transfected with shRNA plasmids and selected for six days with puromycin. B) Cells were co-transfected with a shRNA plasmid and human *Mbd3* or control plasmid and selected with both puromycin and G418 for five days. The target sequences in *Mbd3* shRNA1 and 2 do not exist in human *Mbd3* sequence. C) Chromatin immunoprecipitation (ChIP) analysis using acetylated histone 3 antibody. CGR8 cells were transfected with shRNA plasmids and selected for five days with puromycin. Various pairs of PCR primer were designed to scan respective promoters. Gene expressions were normalized to internal control *β* -actin and presented as the fold induction relative to control samples. Error bars in panel A, B and C represent standard deviation from three technical repeats.

Although mouse ES cells can give rise to all types of cells in an embryo, they can only differentiate directly into three cell lineages: trophectoderm, primitive endoderm and primitive ectoderm (reviewed in [41]). The observed morphological changes after knockdown of *Mbd3* in ES cells did not clearly indicate into which lineages they may have differentiated. Therefore, we analyzed specific molecular markers for the three cell lineages in ES cells after *Mbd3* knockdown. Quantitative RT-PCR analysis confirmed that Mbd3 RNA levels were decreased to about twenty five percent of controls. Noticeably, the trophectoderm markers *Cdx2, Eomes* and *Hand1* were upregulated dramatically in *Mbd3* shRNA cells (Figure 2A). Transcription of primitive endoderm markers, *Gata4* and *Hnf4* (Figure 2A), *Sox7, tPA* and *AFP* (data not shown) did not show obvious changes. Expression of *Gata6*, which has been used as a marker for primitive endoderm, increased at least four fold (Figure 2A). It should be noticed, however, that *Gata6* is also expressed in early trophectoderm [43]. There were no meaningful changes at the RNA level of primitive ectoderm marker *Fgf5* and its derivative mesoderm marker *T*. It appears that knockdown of *Mbd3* promotes ES cells to differentiate towards trophectoderm based on these molecular studies. The differentiation of ES cells into trophectoderm cells is usually accompanied by downregulation of pluripotent genes, such as *Oct4* and *Nanog*. However, we did not observe transcriptional changes for *Oct4, Nanog* and *Esrrb* (Figure 2A), suggesting the observed upregulation of trophectoderm genes may not be a sufficient indicator of a full commitment to the trophectoderm lineage.

To test the specificity of the *Mbd3* RNA interference effect on ES cells, we co-transfected human *Mbd3* and mouse *Mbd3* shRNA plasmids into ES cells and attempted to rescue the observed upregulation of trophectoderm markers. Human Mbd3 and mouse Mbd3 exhibit 95.8% identity at the amino acid level based on protein alignment. Human *Mbd3* cannot be targeted by either mouse *Mbd3* shRNAs as judged by significant DNA sequence divergence. Quantitative RT-PCR results indicated that human *Mbd3* clearly rescued expression of the trophectoderm markers to control levels (Figure 2B). To rule out that what we observed was unique to CGR8 ES cells, we repeated the *Mbd3* RNAi experiment in another mouse ES cell line, R1. Unlike CGR8, R1 cells are usually maintained on a mouse embryonic fibroblast feeder layer. Similar to CGR8 cells, expression of trophectoderm specific genes in R1 cells was increased upon *Mbd3* downregulation; however, the morphological changes were less obvious (data not shown).

Since the NuRD complex is involved in histone deacetylation, we further examined the histone acetylation status of those genes that showed upregulation upon *Mbd3* knockdown. By scanning promoter regions of *Cdx2, Eomes* and *Gata6* using chromatin immunoprecipitation (ChIP) coupled with quantitative PCR analysis, we uncovered specific regions in these promoters that show significantly higher acetylated histone 3 modification in *Mbd3* shRNA cells compared with the control (Figure 2C). High levels of histone 3 acetylation in a promoter are usually correlated with active gene transcription [44], [45]. Thus the ChIP results are consistent with the upregulated transcription of these genes. Together, our data suggest that *Mbd3* expression is critical to suppress TE lineage specific gene expression in mouse ES cells.

### Suppression of *Mbd3* expression is not sufficient for ES cell differentiation to TE lineage

Since *Mbd3* suppression results in ES cell morphological changes and upregulated trophectoderm associated gene expression, we next set out to examine whether compromised *Mbd3* expression can cause definitive trophectoderm differentiation. We carried out a chimeric embryo assay in which cells to be tested are fluorescently labeled and aggregated with early wild type embryos. By checking distributions of the labeled cells in the chimeras, one can assign differentiation lineages to the cells [46], [47].

**Figure 3:**
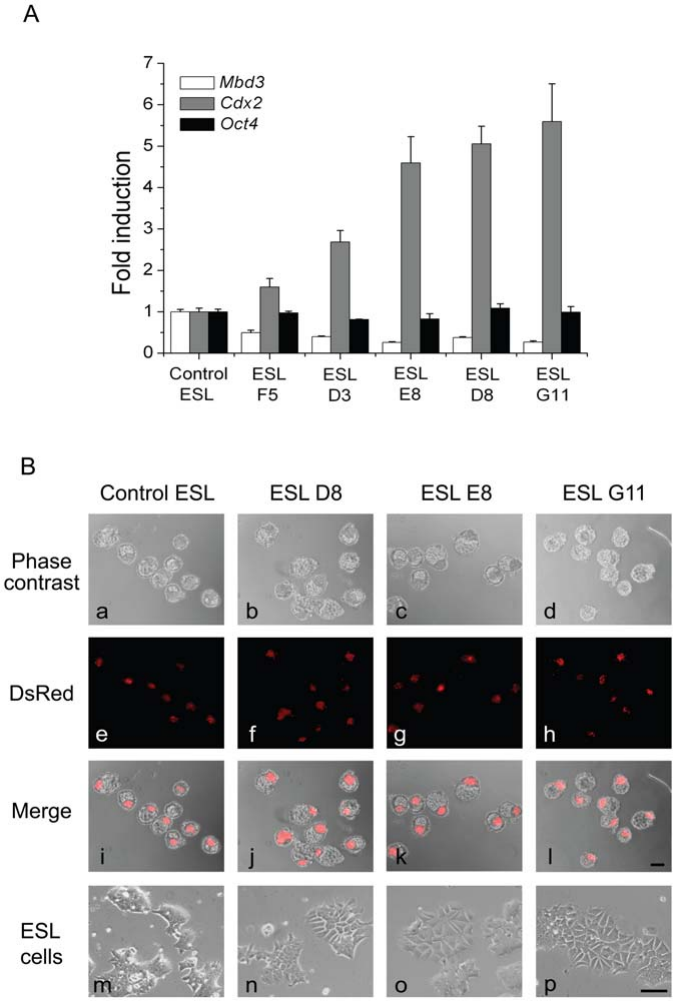
Knockdown *Mbd3* is not sufficient for ES cell differentiation. A) Quantitative RT-PCR analysis of *Cdx2* and *Oct4* in *Mbd3* knockdown stable lines. Control ESL represents control siRNA stably transfected mouse ES cells; ESL F5, ESL D3 ESL E8, ESL D8, and ESL G11 represent five independent Mdb3 siRNA stably transfected mouse ES cells. Error bars represent standard deviation from three technical repeats. B) Chimeric analysis of control and three independent siRNA ES cells in wild type mouse embryos. siRNA stably trasnfected cells were aggregated with wild type mouse embryos at the eight-cell stage and cultured to the blastocyst stage (a–l). in vitro morphology of the siRNA ES cells used in aggregations (m–p). Aggregated cells marked by DsRed. Scale bars: l, 200μm (also applies to a to k); p, 100μm (also applies to m, n, o).

**Figure 4:**
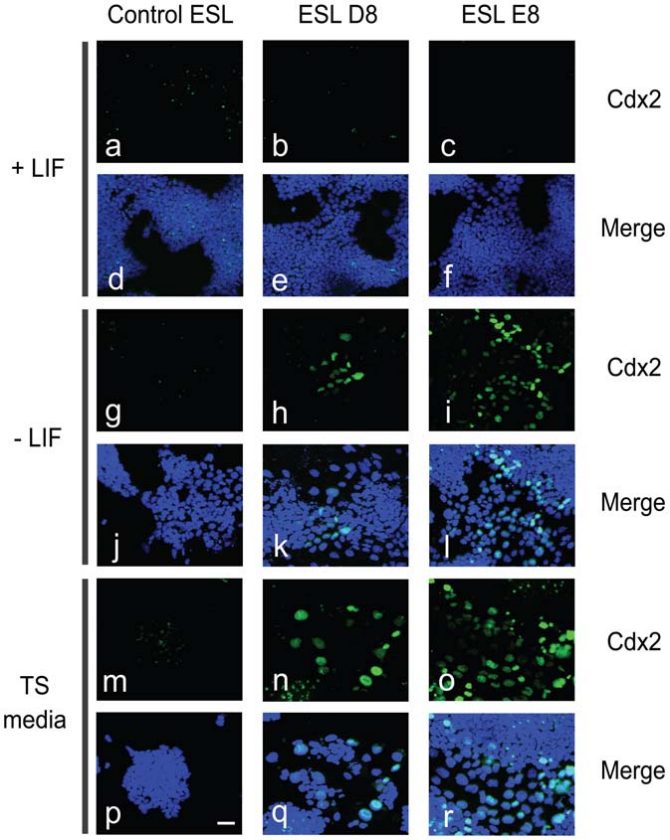
Withdrawal of LIF or cultured under TS cell condition promotes Mbd3 knockdown ES cells to differentiate towards trophectoderm lineage. Cdx2 staining of control or Mbd3 knockdown stable cells with or without LIF, or cultured under TS culture condition. (a–f) Cells were cultured in normal ES cell condition with LIF; (g–l) Cells were withdrawal of LIF for 4 days; (m–r) Cells were cultured under TS culture condition for 6 days. Hoechst stains the cell nuclei. The scale bar represents 50μm.

Transient transfection might result in loss of plasmids during the chimera development, and thus could make it difficult to interpret the differentiation potential of labeled *Mbd3* shRNA cells. To circumvent this drawback of transient transfection for in vivo assays, we made stable *Mbd3* knockdown ES cell lines by lentivirus infection. These cell lines show consistent and stable *Mbd3* mRNA knockdown during the course of the study (Figure 3A), thus avoiding any significant phenotypic variations possible with transient cell knockdowns. Among the *Mbd3* knockdown stable lines, the upregulation of *Cdx2* is inversely correlated with *Mbd3* RNA level. Consistent with the previous transient knockdown result, the expression level of pluripotency marker *Oct4* shows no obvious change among the stable lines (Figure 3A). We also observed that these cells showed fibroblast-like morphology with loose cell-cell contacts (Figure 3B–n, o, p). We aggregated cells from three independent *Mbd3* knockdown stable lines (ESL D8, E8 and G11) and wild type mouse embryos to form chimeras. We examined which cell lineage the *Mbd3* stable cells can associate with. *Mbd3* cells, including those cell lines with obvious differentiated morphology (ESL G11), were shown to integrate into the ICM of chimeric embryos in almost all cases (Figure 3B). At a minimum, this observation suggests that *Mbd3* shRNA cells and ICM cells share similar cell surface molecules essential for cell sorting. In previous studies [32], *Oct4* and *Nanog* expression changes little in *Mbd3* shRNA cells, which may partially explain why these cells remain associated with the ICM in the chimera embryos (Figure 3B). Moreover, we failed to detect Cdx2 protein expression in these cells (Figure 4b, c), although *Cdx2* mRNA levels were consistently upregulated many fold (Figure 3A), suggesting that either there is not sufficient mRNA transcription or that translational regulation plays a role in these cells. Taken together, these results indicate that reduction of *Mbd3* expression is not sufficient for fully committed TE lineage differentiation.

### *Mbd3* is required for maintaining full differentiation potential of ES cells

Although the *Mbd3* knockdown ES cells retain the ability to remain associated with the ICM, whether they retain ES cell like differentiation capability is not known. Previously, *Mbd3* ^−/−^ cells were shown to retain self-renewal capability upon withdraw of LIF and also to show defective differentiation potential [32]. However, which specific early lineages are compromised is unclear for *Mbd3* ^−/−^ cells.

**Figure 5:**
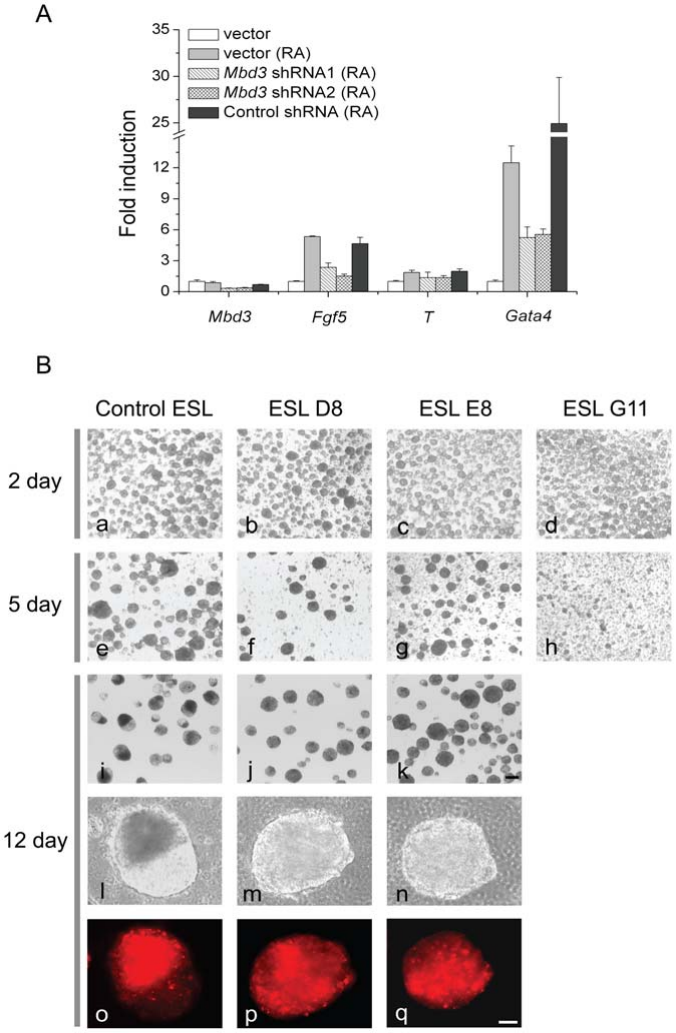
Full differentiation potential of mouse of ES cells is compromised when Mbd3 expression is reduced. A) ES cells cultured with or without retinoic acid for three days were subjected to real-time RT-PCR analysis. For differentiations, 1μM retinoic acid (RA) was used or LIF was withdrawn from the medium. ES cells were transfected with respective plasmids for three days before RA addition and selected with antibiotics for five days. Error bars represent standard deviation from three technical repeats. B) Embryoid body (EB) formation of control or Mbd3 knockdown stable cells. Stable cells (2×105) were suspension cultured in ES cell medium without LIF in 35 mm cell dishes. Scale bars: k, 200μm (also applies to a to j); q, 50μm (also applies to l to p).

If *Mbd3* shRNA cells show upregulated trophectodermal markers, an indication of greater potential for re-specification towards the trophectoderm lineage, we reasoned their capability to form primitive ectoderm, precursor of three germ layers, might be compromised. To investigate this possibility, we tried to induce *Mbd3* shRNA-transduced cells to differentiate by adding retinoic acid (RA, all-trans), an agent which causes wild type ES cells to convert into cells comprising all three germ layers [48]. Compared with control shRNA cells, *Mbd3* knockdown cells showed reduced induction of *fgf5* (ectoderm) and *gata4* (endoderm) (Figure 5A). This result indicates *Mbd3* indeed is required for full differentiation potential of primitive ectoderm layers.

We also used embryoid body (EB) formation by suspension culture of ES cells to examine their differentiation capability. Successfully differentiated EB is a three-dimensional spheroid structure that mimics post-implantation embryos and contains three germ layers [49]. Since *Mbd3* seems to regulate ES cell differentiation, it is possible that reduction of *Mbd3* expression in ES cells may also cause abnormal differentiation in EB. We used three separate *Mbd3* knockdown cell lines (ESL D8, E8 and G11) in the assay. All cell lines except ESL G11 form aggregates by day five (Figure 5B–e, f, g). By day twelve, in contrast to control cells which form heterogeneous spheroids in the aggregates, ESL D8 and ESL E8 cells only show aggregated solid “cell balls’ similar to those at day five, suggesting the lack of robust differentiation shown by control ES cells (Figure 5B, i–q). Moreover, even the ESL G11 cell line with the most severe differentiation morphology failed to generate obvious EB by day five, suggesting severely compromised differentiation capability (Figure 5B–h). Before day twelve, the remaining small ESL G11 cell aggregates are all disintegrated (data not shown). It is clear that *Mbd3* is required for the formation of normal EB, another indication of full differentiation potential. Together, these results strongly suggest that *Mbd3* is essential for mouse ES cells to maintain full pluripotency.

### *Mbd3* reduced ES cells are prone to differentiate into trophectoderm lineage

*Mbd3* knockdown cells showed substantial increased mRNA expression of trophectoderm lineage markers, a strong indication of trophectoderm differentiation. However, we did not detect Cdx2 protein expression in *Mbd3* knockdown cells (Figure 4b, c). These cells were shown to integrate into the ICM of chimera embryos (Figure 3) which indicates no definitive differentiation to trophectoderm lineage at least in the chimeras. Although we can not rule out that the *Mbd3* knockdown cells have reverted to a more pluripotent state by in vivo factors when placed in the mileu of a developing embryo, it is also possible that reduction of *Mbd3* in ES cells may reduce the intrinsic threshold for these cells to differentiate towards TE lineage.

Leukemia inhibitory factor (LIF) is one of the key components in suppression of spontaneous differentiation for mouse ES cells. Withdrawal of LIF from the culture medium allows ES cells to differentiate randomly into multiple lineages [50]. However, mouse ES cells rarely differentiate into trophectoderm in various culture conditions. Alterations of Cdx2 and Eomes were reported to trigger trophectoderm differentiation of mouse ES cells [34], [51]. We attempted to test whether *Mbd3* knockdown cells have a tendency to differentiate into trophectoderm in the absence of LIF. After removal of LIF from the culture medium for 4 days, we detected Cdx2 protein expression in the nuclei of *Mbd3* shRNA cells, but not in the control cells (Figure 4). These results indicate that *Mbd3* knockdown cells seem to be biased to the trophectoderm lineage.

**Figure 6:**
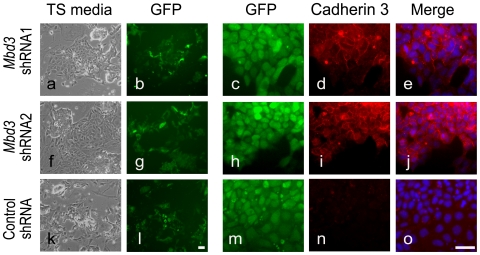
Reduction of Mbd3 expression in mouse ES cells promotes trophectoderm stem (TS) cell derivation. Mouse ES cells transfected with contro or Mbd3 shRNAs for three days were cultured in trophoblast stem cell medium to promote differentiation into TS cells. GFP expression was used to monitor shRNA plasmids transfection (b, c, g, h, l, and m). Hoechst stains the cell nuclei. Red fluorescence shown on cell boundaries indicates the expression of cadherin 3, a surface antigen specific to trophoblast cells (d, e, i, j). Scale bars: l, 50μm (also applies to a, b, f, g, k); o, 50μm (also applies to c–e, h–j, m–n).

Previously, overexpression of Cdx2 in mouse ES cells was shown to induce trophectoderm differentiation, likely by directly inhibiting Oct4 functions [34], [52]. When ES cells are induced to differentiate toward trophectoderm, trophoblast stem (TS) cells can be derived from these cells in appropriate culture conditions [34], [47]. To further confirm that *Mbd3* knockdown cells are indeed biased towards trophectoderm differentiation, we cultured control and *Mbd3* shRNA stable cells under TS culture conditions containing FGF4 and MEF-conditioned medium [40] for 6 days. Although we did not observe representative TS colonies, the Cdx2 proteins were detected in *Mbd3* shRNA stable cells (Figure 4n, o). Moreover, with prolonged culture of *Mbd3* knockdown cells three days after shRNAs transfection in TS cell medium, we observed formation of flattened TS-like colonies by passage two (Figure 6a, f) which expressed trophectoderm cell surface marker Cadherin3 (Cdh3, also known as placenta Cadherin) (Figure 6d, i). In contrast, there were no Cadherin3 positive cells in control shRNA cells under the same culture condition (Figure 6n). It is likely that both TS culture condition and withdrawal of LIF further promote *Mbd3* shRNA cells towards a trophectoderm lineage. These results suggest *Mbd3* helps mouse ES cells maintain pluripotency by partial suppression of the trophectoderm lineage.

## Discussion

The NuRD complex exists as a co-repressor of gene expression in a broad range of different cells [23–27; 30–31]. In this study, we selectively reduced the expression of *Mbd3* and further investigated its epigenetic functions in mouse ES cells. We find that reduction of *Mbd3* expression lowers the threshold of ES cells to differentiate towards the trophectoderm lineage. Our results thus indicate that *Mbd3* is essential to maintain full mouse ES cell pluripotency by helping repress the trophectoderm specific differentiation program.

Trophectoderm specification is the very first cellular differentiation of early mouse embryos. The upregulation of Cdx2 expression has been shown to be important for the formation of trophectoderm [35]. In addition, Oct4 is normally downregulated in trophectoderm cells in vivo and in vitro. Cdx2 and Oct4 also antagonize each other at the transcription level [34], [35]. However, knockdown of *Mbd3* in mouse ES cells upregulates *Cdx2* RNA while *Oct4* RNA remains unchanged (Figure 2A, 3A). Similarly, Oct4 expression was previously shown to be unchanged in *Mbd3* ^−/−^ ES cells [32]. Lack of *Cdx2* expression data in *Mbd*3^−/−^ ES cells precludes direct comparison of Cdx2 expression in the two studies. Several observations may be used to explain why Oct4 remains constant in *Mbd3* compromised cells. First, *Oct4*-null ES cells with constitutive expression of transfected Oct4 and overexpression of Cdx2 were differentiated into TE lineage, suggesting expression of Oct4 alone is not sufficient for blocking trophectoderm differentiation triggered by Cdx2 overexpression [34]. Second, Oct4 downregulation during normal trophectoderm differentiation may require epigenetic regulations, which may be positively influenced by the NuRD complex. In fact, Gu and colleagues recently found that Mbd3 was recruited by GCNF to the *Oct4* promoter to repress its expression through DNA methylation in the process of ES cell differentiation [53]. Thus loss of *Mbd3* in ES cells may lead to deregulation of *Oct4* transcription during ES cell differentiation. Furthermore, in the *Mbd3* knockdown cells, Cdx2 protein was not appreciably increased, indicating that alteration of *Mbd3* mRNA level may not significantly alter Cdx2 translation.

The morphological change and expression of trophectoderm genes in *Mbd3* knockdown cells (Figure 1D, 2, 3A, and 3B m–p) are considered as early indicators of TE differentiation, but further chimera analysis (Figure 3B a–l) suggest that these cells have properties that resemble those of ICM. However, further subjection of *Mbd3* knockdown cells to differentiation challenges, such as withdrawal of LIF or TS culture medium resulted in Cdx2 protein expression, indicating that *Mbd3* knockdown cells are biased to differentiation. In contrast, *Mbd3^−/−^* ES cells could be maintained in the absence of LIF. The discrepancy between these two studies appears to be significant, but it should be emphasized that in both cases disruption of Mbd3 function did not affect cell proliferation and only affected some differentiation potential. We showed that in the absence of LIF, *Mbd3* shRNA cells express Cdx2 protein, which might explain why trophoblast markers *Tpbpa* and *Pl-1* were observed in *Mbd3^−/−^* ES cells ([32] and Figure 2A). It would be interesting to test whether *Mbd3^−/−^* ES cells are also biased towards TE lineage. It is surprising that Cdx2 protein did not shown increased expression upon *MBD3* knock down despite strong upregulation of its mRNA. It is possible that translation efficiency of elevated Cdx2 mRNA is negatively controlled by unknown mechanisms, such as micro RNAs. Future experiments may help us elucidate the discrepancy between mRNA and protein expression.

In the embryoid body formation assay, the *Mbd3^−/−^* ES cells showed restricted differentiation potential, and this was correlated with upregulation of trophoblast markers *Tpbpa* and *Pl-1* and primitive ectoderm marker *Fgf5*. The embryoid body formation result using *Mbd3* knockdown cells is consistent with that of *Mbd3^−/−^* ES cells. *Mbd3* shRNA cells are also partially resistant to retinoic acid induced differentiation towards embryonic ectoderm, mesoderm and endoderm cells (Figure 4A). We reason that *Mbd3* shRNA cells may have undergone necessary, albeit not fully sufficient, changes towards trophectoderm lineage even though their morphological changes are obvious. This may also explain why *Mbd3^−/−^* ES cells and *Mbd3* shRNA cells fail to differentiate normally upon RA addition or in EB formation ([32], Figure 5). Therefore, it would be interesting to examine expression of early trophectoderm markers in *Mbd3^−/−^* ES cells in addition to markers of mature trophectoderm. Our results also raise an interesting point in that morphological change alone, in certain ES cell lines, cannot be used to judge their differentiation potentials. The reason why *Mbd3* reduction introduces differentiation bias towards trophectoderm lineage, but not definite commitment is worthy of further investigation.

Investigation of how the NuRD complex regulates target genes in ES cells should help elucidate epigenetic mechanisms in maintaining ES cell pluripotency. Understanding what and how NuRD complex components are assembled in ES cells and how they function during lineage specific differentiations will be helpful in understanding epigenetic controls in general.

## Supporting Information

Table S1 Primer sequences used in quantitative PCR
(0.05 MB DOC)

Table S2 Primer sequences used in ChIP
(0.04 MB DOC)
