# Supplementary material for: Mbd3, a Component of NuRD/Mi-2 Complex, Helps Maintain Pluripotency of Mouse Embryonic Stem Cells by Repressing Trophectoderm Differentiation
Source: PLoS One. 2009 Nov 3;4(11):e7684. doi: 10.1371/journal.pone.0007684 (PMC2766630; doi:10.1371/journal.pone.0007684)
Supplement: Table S1 — Primer sequences used in quantitative PCR (0.05 MB DOC) [file pone.0007684.s001.doc]

Table S1. Primer sequences used in quantitative PCR

| Gene symbol | Primer sequence |
| --- | --- |
| *β-actin^＊^* | Up: 5-AGTGTGACGTTGACATCCGTA-3 |
|  | Down: 5-GCCAGAGCAGTAATCTCCTTCT-3 |
| mouse *Mbd3^＊^* | Up: 5-CCCCAGCGGGAAGAAGTTC-3 |
|  | Down: 5-CGGAAGTCGAAGGTGCTGAG-3 |
| *Chd4^＊^* | Up: 5-GAAATTGCTGCGGCACCATTA-3 |
|  | Down: 5-AGCCATCATTGTAGTTGACCTG-3 |
| *Oct4* | Up: 5- CTCCCTACAGCAGATCACTCACA -3 |
|  | Down: 5- GAACCATACTCGAACCACATCCT -3 |
| *Nanog^＊^* | Up: 5-TTGCTTACAAGGGTCTGCTACT-3 |
|  | Down: 5-ACTGGTAGAAGAATCAGGGCT-3 |
| *Esrrb* | Up: 5-TTCTCATCTTGGGCATCGTGTA-3 |
|  | Down: 5-CTCGGCGTATGCCAGCTT-3 |
| *Cdx2^＊^* | Up: 5-AGACAAATACCGGGTGGTGTA-3 |
|  | Down: 5-CCAGCTCACTTTTCCTCCTGA-3 |
| *Eomes* | Up: 5-GAGCTTCAACATAAACGGACTCAA-3 |
|  | Down: 5-CGGCCAGAACCACTTCCA-3 |
| *Hand1* | Up: 5-TGCGCCTGGCTACCAGTTA-3 |
|  | Down: 5-GGGTCACCTGCTTGTGCAT-3 |
| *Gata6* | Up: 5- TCAGGTCAAGACGGCCTCTAC -3 |
|  | Down: 5- TCGCACGGAGGATGTGACT -3 |
| *Gata4* | Up: 5-CGGGCCAACCCTGGAA-3 |
|  | Down: 5-TTGACACACTCTCTGCCTTCTGA-3 |
| *Hnf4* | Up: 5-CAAGAACACATGGGCACCAA-3 |
|  | Down: 5-ATCTGTCCATTGCTGAGGTGAGA-3 |
| *Fgf5* | Up: 5-AGAGTGGGCATCGGTTTCC-3 |
|  | Down: 5-GCTTCGTGGGAGCCATTG-3 |
| *Brachyury (T)* | Up: 5-ACAGATCATGTTAAACTCCTTGCATAA-3 |
|  | Down: 5-GCGGGCCCCCAACTC-3 |
| human *Mbd3* | Up: 5-ggccacagggatgtctttta -3 |
|  | Down: 5-Ttgacctggttggaggagtcg -3 |
