# Supplementary material for: Mbd3, a Component of NuRD/Mi-2 Complex, Helps Maintain Pluripotency of Mouse Embryonic Stem Cells by Repressing Trophectoderm Differentiation
Source: PLoS One. 2009 Nov 3;4(11):e7684. doi: 10.1371/journal.pone.0007684 (PMC2766630; doi:10.1371/journal.pone.0007684)
Supplement: Table S2 — Primer sequences used in ChIP (0.04 MB DOC) [file pone.0007684.s002.doc]

Table S2. Primer sequences used in ChIP

| Gene symbol | Primer sequence |
| --- | --- |
| *β-actin* | Up: 5-GCCTGACTCCATGAGAAGTTTTGT-3 |
|  | Down: 5-CCCTGCCCCGGATCTAGA-3 |
| *Oct4-1* | Up: 5-GCAGTGCCAACAGGCTTTGT-3 |
|  | Down: 5-GGTCACCGGACACCTCACA-3 |
| *Oct4-2* | Up: 5-GGTCCCGTCCTAAGGGTTGT-3 |
|  | Down: 5-GAGCGCTATCTGCCTGTGTCT-3 |
| *Cdx2-1* | Up: 5-GGATCCCAGCCATCCACTAAT-3 |
|  | Down: 5-CCCTCTTCAAAGCCAACAACTG-3 |
| *Cdx2-2* | Up: 5-GAGGTTAAAGTGCACCCAGGTT-3 |
|  | Down: 5-TTCAGGCCCTTCTTGCTAGCT-3 |
| *Cdx2-3* | Up: 5-CAGCCATTGGTGTCTGTGTCAT-3 |
|  | Down: 5-CCAGCGGCCTTACGTGAT-3 |
| *Eomes-1* | Up: 5-TATGCCGTGGGTAGACCATGT-3 |
|  | Down: 5-TCTATGGCGCCGGAGAAAC-3 |
| *Eomes-2* | Up: 5-CCCTGGCCGCACATATATAAG-3 |
|  | Down: 5-GGCATACTTGACCGCTTGGA-3 |
| *Gata6-1* | Up: 5-CCCCAGCACCTCATAGTTTCAA-3 |
|  | Down: 5-TCAGAACAACATTCCCCAGCTA-3 |
| *Gata6-2* | Up: 5-ACCTGAGCATCCCGAAACAG-3 |
|  | Down: 5-TTAAGCCCCGCGCAGTT-3 |
| *Gata6-3* | Up: 5-GGATCCCTCCTCCTTCTCTTCA-3 |
|  | Down: 5-CAGGCTGTGGGTCGGAACT-3 |

**“＊”** denotes quantitative PCR primers adapted from PrimerBank (<http://pga.mgh.harvard.edu/primerbank/index.html>) [54]

The rest of the primers were designed using Primer Express software fit for ABI 7900 quantitative PCR machine.
